# Giant cell arteritis-related cerebrovascular ischemic events: a French retrospective study of 271 patients, systematic review of the literature and meta-analysis

**DOI:** 10.1186/s13075-023-03091-x

**Published:** 2023-07-07

**Authors:** Thomas Penet, Marc Lambert, Clio Baillet, Olivier Outteryck, Hilde Hénon, Sandrine Morell-Dubois, Eric Hachulla, David Launay, Mohammad Ryadh Pokeerbux

**Affiliations:** 1 Univ. Lille, Inserm, CHU Lille, Service de Médecine Interne Et Immunologie Clinique, Centre de Référence Des Maladies Autoimmunes Systémiques Rares du Nord Et Nord-Ouest de France (CeRAINO), U1286 - INFINITE - Institute for Translational Research in Inflammation, 59000 Lille, France; 2 Service de Médecine Interne et Immunologie Clinique, CHU Lille, Rue Michel Polonovski, F-59037 Lille Cedex, France; 3 Nuclear Medicine Department, CHU Lille, Lille, France; 4 Department of Neuroradiology, Univ. Lille, INSERM, CHU Lille, U1172 – Lille Neurosciences Institute, 59000 Lille, France; 5 Univ. Lille, Inserm, CHU Lille, U1172 - LilNCog-Lille Neuroscience & Cognition, Lille, France

**Keywords:** Giant cell arteritis, Cerebrovascular ischemic events, Prevalence, Risk factors, Imaging, Meta-analysis

## Abstract

**Background:**

Cerebrovascular ischemic events (CIE) are among the most severe complications of giant cell arteritis (GCA). Heterogeneity between different studies in the definition of GCA-related CIE leads to uncertainty regarding their real prevalence. The aim of our study was to evaluate the prevalence and describe the characteristics of GCA-related CIE in a well-phenotyped cohort completed by a meta-analysis of the existing literature.

**Methods:**

In this retrospective study performed in the Lille University Hospital, all consecutive patients with GCA according to American College of Rheumatology (ACR) diagnostic criteria were included from January 1, 2010, to December 31, 2020. A systematic review of the literature using MEDLINE and EMBASE was performed. Cohort studies of unselected GCA patients reporting CIE were included in the meta-analysis. We calculated the pooled summary estimate of GCA-related CIE prevalence.

**Results:**

A total of 271 GCA patients (89 males, mean age 72 ± 9 years) were included in the study. Among them, 14 (5.2%) presented with GCA-related CIE including 8 in the vertebrobasilar territory, 5 in the carotid territory, and 1 patient having multifocal ischemic and hemorrhagic strokes related to intra-cranial vasculitis. Fourteen studies were included in the meta-analysis, representing a total population of 3553 patients. The pooled prevalence of GCA-related CIE was 4% (95% CI 3–6, *I*^*2*^ = 68%).

Lower body mass index (BMI), vertebral artery thrombosis on Doppler US (17% vs 0.8%, *p* = 0.012), vertebral arteries involvement (50% vs 3.4%, *p* < 0.001) and intracranial arteries involvement (50% vs 1.8%, *p* < 0.001) on computed tomography angiography (CTA) and/or magnetic resonance angiography (MRA), and axillary arteries involvement on positron emission computed tomography (PET/CT) (55% vs 20%, *p* = 0.016) were more frequent in GCA patients with CIE in our population.

**Conclusions:**

The pooled prevalence of GCA-related CIE was 4%. Our cohort identified an association between GCA-related CIE, lower BMI, and vertebral, intracranial, and axillary arteries involvement on various imaging modalities.

**Supplementary Information:**

The online version contains supplementary material available at 10.1186/s13075-023-03091-x.

## Introduction

Giant cell arteritis (GCA) is the most common form of vasculitis in patients over 50 years of age. This granulomatous vasculitis of large and medium arteries affects the aorta and its major branches, with a predilection for the external carotid and ophthalmic arteries, and to a lesser extent the vertebral arteries [1–3].

Among the most serious complications of GCA are ischemic events, mainly ophthalmologic complications, and cerebral ischemic complications. Both ophthalmic and cerebral ischemic complications occur mostly early in the course of GCA and can occur despite initiation of corticosteroid therapy [4]. Studies conducted prior to the use of corticosteroids to treat GCA reported a high prevalence of ophthalmologic ischemic complications (35–60%), while the prevalence is lower (12–20%) in studies conducted since the use of corticosteroids [2, 3].

Cerebrovascular ischemic events (CIE) are more rarely associated with GCA. In a 2016 meta-analysis, Ungprasert et al. found a pooled risk ratio of cerebrovascular events of 1.40 (95% CI 1.27–1.56) in patients with GCA versus non-GCA comparators [5]. The incidence of CIE in GCA has been estimated at 0.76/100,000 patient-years in a 2015 French study by Samson et al. [6]. The reported prevalence of CIE ranges from 1.5 to 12.95% [2, 6–21]. This heterogeneity relies partly on the different definition of GCA-related CIE.

While the risk factors for ophthalmic ischemic complications have been well studied, these are less well known in the case of GCA-related CIE. Some studies have used both ophthalmic and cerebral ischemic complications as a combined endpoint [7–9, 13, 19, 21–25], while others have specifically evaluated risk factors for CIE [4, 10, 11, 14–18, 20, 26], which leads to variable results.

The aim of the present study was to fill these gaps by evaluating the prevalence of GCA-related CIE in a well phenotyped cohort of GCA patient and by a meta-analysis of the existing literature and to characterize these patients.

## Patients and methods

### French cohort study

#### Population

We conducted a retrospective observational study in the Internal Medicine Department of Lille University Hospital in France (National Center for Systemic Autoimmune Diseases). Medical Information Department was queried with relevant International Classification of Diseases, 10th Edition (ICD-10) codes, in order to screen all patients having been diagnosed with GCA between January, 1 2010, and December, 31 2020, and among them, those who presented with CIE (established or transient strokes). The ICD-10 codes used were M316 (giant cell arteritis), I63 (cerebral infarct), I64 (stroke, not specified as hemorrhagic or infarct stroke), and G45 (transient stroke and related syndromes). Patients identified with the ICD-10 codes were included if they met American College of Rheumatology (ACR) 1990 diagnostic criteria [27]. Among the patients screened with GCA who presented with one or more CIE, medical records were reviewed to assess whether the CIE were GCA-related or not.

A CIE was defined as GCA-related if it was clearly linked to GCA at diagnosis or relapse, within a delay of 4 weeks from diagnosis or relapse, after reviewing patients’ medical records, and in the absence of another well-identified etiology (mainly atherosclerosis, embolic or cerebral small vessel disease).

Ischemic vascular events during follow-up were defined by the occurrence of non-GCA related CIE, myocardial infarction, or limb ischemia.

#### Data collection

The following data were collected at baseline: patient demographics, vascular risk factors, vascular history, atrial fibrillation, GCA symptoms, polymyalgia rheumatica symptoms, inflammatory biological markers, temporal artery biopsy (TAB) findings, ischemic complications, inflammatory vascular involvement as diagnosed on Doppler ultrasound (Doppler US) (mainly hypoechoic wall thickening and vertebral artery thrombosis), positron emission computed tomography (PET/CT) (vascular hypermetabolism), computed tomography angiography (CTA) (including cervico-thoraco-abdominal CTA and brain CTA), and brain magnetic resonance angiography (MRA). During follow-up, specific treatment was also recorded as well as ischemic vascular events.

### Systematic review of the literature and meta-analysis

The meta-analysis was conducted according to MOOSE guidelines. MEDLINE and EMBASE databases were queried by two of the authors (TP and MRP) using the following search terms: (giant cell arteritis, horton [MeSH Terms]) AND (cerebrovascular accident [MeSH Terms]) and ‘giant cell arteritis’/exp AND ‘cerebrovascular accident’/exp.” All records published before October 22, 2022, were included in the search. Language was restricted to English or French. Reference list of selected studies was hand-searched for additional relevant studies to be included in the meta-analysis.

Two of the authors (TP and MRP) independently screened the titles and abstracts of the retrieved records to identify eligible articles to be studied in full-text. The two reviewers then read the full-text of eligible articles for inclusion in the meta-analysis. Selected articles were compared and, in case of disagreement, decisions were made by consensus. Studies of unselected adult GCA patients, assessing a CIE prevalence, were included in the analysis. Studies from a specific center were included if their respective study periods were different. If for a same center, two studies covered an overlapping study period, data from the largest cohort were kept.

Quality of the studies was assessed using the Joanna Briggs Institute (JBI) Critical Appraisal Checklist for studies reporting prevalence data [28].

Data were extracted and entered into a predefined spreadsheet table which included the following items: name of the first author, title of the study, year of publication, country, study design, study period, GCA diagnosis criteria, ischemic complications studied, study population, the definition, number, and prevalence of GCA-related CIE.

### Statistical analysis

Continuous variables were expressed as means ± standard deviation (SD) or medians (interquartile range (IQR)). Categorical variables were expressed as number (%). Comparisons between groups were conducted using Fisher’s exact test or Pearson’s chi-squared test, as appropriate, for categorical variables, and Wilcoxon rank-sum test for continuous variables. A *p* value of ≤ 0.05 defined statistical significance.

For the meta-analysis, we calculated a weighted pooled summary estimate of proportion of GCA-related CIE. Proportions of individual studies was transformed using Freeman-Tukey double arcsine method for normalizing and variance-stabilizing the sampling distribution. For the meta-analysis, a random-effects model estimated with the DerSimonian and Laird method was used. Accordingly, studies were considered to be a random sample from a population of studies. Heterogeneity was assessed using an *I*^2^ statistic and a chi-square heterogeneity statistic. The overall effect was estimated using a weighted average of individual effects, with weights inversely proportional to variance in observed effects.

Meta-regression was used to assess the impact of delay from diagnosis to define a CIE to be GCA-related.

All analyses were performed using R with meta and metafor packages; *p* values less than 0.05 were considered significant.

## Results

### French cohort study

#### Baseline characteristics

Of the 339 patients screened, 271 (33% men) met the ACR diagnostic criteria and were therefore included in the present study (Table 1). Mean age at the diagnosis was 72 ± 9 years. Median follow-up time was 48 months (21–81). TAB was positive in 137 (55%) out of 248 patients who underwent it. Vascular risk factors were present in 207 (76.4%) patients, mainly arterial hypertension (56%), and 35 (13%) patients had atrial fibrillation. The most frequent symptom at diagnosis was headache (71%). The median C-reactive protein (CRP) level was 70 mg/l (35–120).

**Table 1:** Comparison of baseline characteristics according to GCA-related CIE

Characteristic	*N*	Overall, *N* = 271	No GCA-related CIE, *N* = 257	GCA-related CIE, *N* = 14	*p*-value
**Age at diagnosis (years)**	271	72 ± 9	72 ± 9	70 ± 9	0.3
**Follow-up (months)**	271	48 (21–81)	50 (22–84)	24 (9–31)	**0.02**
**Vascular risk factors**	271	207 (76.4%)	197 (76.6%)	10 (71.4%)	NS
Male	271	89 (33%)	81 (32%)	8 (57%)	0.075
Hypertension	271	151 (56%)	145 (56%)	6 (43%)	0.3
Diabetes	271	54 (20%)	51 (20%)	3 (21%)	> 0.9
Dyslipidemia	271	99 (37%)	94 (37%)	5 (36%)	> 0.9
Smoking	267	83 (31%)	76 (30%)	7 (54%)	0.12
BMI (kg/m^2^)	147	24.8 (22.1–28.4)	24.9 (22.3–28.6)	22.5 (18.0–24.1)	**0.02**
**Vascular history**					
Coronary heart disease	271	11 (4.1%)	11 (4.3%)	0 (0%)	> 0.9
Stroke history	271	24 (8.9%)	23 (8.9%)	1 (7.1%)	> 0.9
Peripheral artery disease	271	20 (7.4%)	19 (7.4%)	1 (7.1%)	> 0.9
**Atrial fibrillation**	271	35 (13%)	33 (13%)	2 (14%)	0.7
**Positive temporal artery biopsy**	248	137 (55%)	129 (55%)	8 (57%)	0.9
**Clinical features**					
Constitutional symptoms	263	150 (57%)	141 (56%)	9 (69%)	0.4
Fever	263	53 (20%)	51 (20%)	2 (15%)	> 0.9
Headache	264	188 (71%)	181 (72%)	7 (54%)	0.2
Jaw claudication	263	104 (40%)	98 (39%)	6 (46%)	0.6
Scalp tenderness	262	99 (38%)	97 (39%)	2 (15%)	0.087
Limb claudication	262	27 (10%)	27 (11%)	0 (0%)	0.4
Non palpable temporal pulse	262	46 (18%)	44 (18%)	2 (15%)	> 0.9
Polymyalgia rheumatica	270	93 (34%)	89 (35%)	4 (29%)	0.8
**Ischemic complications**					
***Cerebrovascular ischemic events***					
Time from diagnosis to GCA related first CIE (days)	14	− 5 (− 22–0)	-	− 5 (− 22–0)	-
GCA-related recurrent CIE	14	2 (0.7%)	-	2 (14%)	-
***Visual involvement***	271	77 (28%)	76 (30%)	1 (7.1%)	0.12
AAION	271	41 (15%)	40 (16%)	1 (7.1%)	0.7
Other visual involvement	271	38 (14%)	38 (15%)	0 (0%)	0.2
***Aortic involvement***	271	9 (3.3%)	8 (3.1%)	1 (7.1%)	0.4
**Biological parameters**					
CRP (mg/l)	251	70 (35–120)	70 (35–120)	44 (24–98)	0.5
Hemoglobin level (g/dl)	169	11.70 (10.60–12.80)	11.65 (10.60–12.80)	12.10 (10.90–13.70)	0.5
**Imagery**					
***Doppler US***					
Temporal artery involvement	250	74 (30%)	70 (29%)	4 (33%)	0.8
Carotid artery involvement	251	43 (17%)	41 (17%)	2 (17%)	> 0.9
Vertebral artery involvement (hypoechogenic thickening)	250	6 (2.4%)	5 (2.1%)	1 (8.3%)	0.3
Thrombosis of vertebral artery	251	4 (1.6%)	2 (0.8%)	2 (17%)	**0.012**
Subclavian artery involvement	251	37 (15%)	35 (15%)	2 (17%)	0.7
Axillary artery involvement	251	43 (17%)	43 (18%)	0 (0%)	0.2
*** PET/CT***					
PET/CT delay from diagnosis (days)	171	1 (− 7–20)	2 (− 7–24)	0 (− 9–0)	0.14
Temporal artery involvement	171	8 (4.7%)	8 (5.0%)	0 (0%)	> 0.9
Carotid artery involvement	171	55 (32%)	51 (32%)	4 (36%)	0.7
Vertebral artery involvement	171	38 (22%)	35 (22%)	3 (27%)	0.7
Subclavian artery involvement	171	85 (50%)	78 (49%)	7 (64%)	0.4
Axillary artery involvement	171	38 (22%)	32 (20%)	6 (55%)	**0.016**
Femoral artery involvement	171	39 (23%)	37 (23%)	2 (18%)	> 0.9
Aortitis	171	92 (54%)	86 (54%)	6 (55%)	> 0.9
Polymyalgia rheumatica	171	36 (21%)	35 (22%)	1 (9.1%)	0.5
*** CTA/MRA***					
CTA/MRA carotid artery involvement	193	31 (16%)	26 (15%)	5 (36%)	0.053
CTA/MRA vertebral artery involvement	193	13 (6.7%)	6 (3.4%)	7 (50%)	** < 0.001**
CTA/MRA axillary artery involvement	172	12 (7.0%)	12 (7.5%)	0 (0%)	> 0.9
CTA/MRA subclavian artery involvement	172	38 (22%)	36 (22%)	2 (17%)	> 0.9
CTA/MRA aortitis	172	72 (42%)	68 (42%)	4 (33%)	0.8
CTA/MRA cerebral artery involvement	71	8 (11%)	1 (1.8%)	7 (50%)	** < 0.001**
CTA cerebral artery involvement	13	4 (31%)	0 (0%)	4 (57%)	0.070
MRA cerebral artery involvement	69	7 (10%)	1 (1.8%)	6 (43%)	** < 0.001**
**Treatments**					
Pulse glucocorticoids	265	71 (27%)	62 (25%)	9 (69%)	**0.001**
Initial oral glucocorticoid dosage (mg/day)	264	55 (45–60)	50 (45–60)	60 (55–80)	**0.017**
Aspirin	270	216 (80%)	204 (80%)	12 (86%)	0.7
Tocilizumab	265	33 (12%)	29 (12%)	4 (29%)	0.081
Methotrexate	270	74 (27%)	72 (28%)	2 (14%)	0.4
**Ischemic vascular events during follow-up**	270	32 (12%)	30 (12%)	2 (14%)	0.7
Ischemic vascular event type	32				> 0.9
Non GCA-related CIE		19 (59%)	17 (57%)	2 (100%)	
Myocardial infarction		9 (28%)	9 (30%)	0 (0%)	
Limb ischemia		4 (12%)	4 (13%)	0 (0%)	
Time from diagnosis to non GCA-related CIE (days)	18	1,499 (717–1,723)	1,378 (626–1,636)	3,073 (2,830–3,316)	0.052
Non GCA-related CIE etiology	18				0.8
Atherosclerosis		2 (11%)	2 (12%)	0 (0%)	
Cardioembolism		6 (33%)	5 (31%)	1 (50%)	
Small vessel disease		3 (17%)	2 (12%)	1 (50%)	
Septic		1 (5.6%)	1 (6.2%)	0 (0%)	
Undetermined		6 (33%)	6 (38%)	0 (0%)	
**Mortality**	271	33 (12%)	29 (11%)	4 (29%)	0.075

Results are expressed as mean ± SD or median (IQR) for quantitative variables, *n* (%) for qualitative variables

*N* Number of patients with available data, *GCA* Giant cell arteritis, *CIE* Cerebrovascular ischemic event, *BMI* Body mass index, *AAION* Acute anterior ischemic optic neuropathy, *CRP* C-reactive protein, *Doppler US* Doppler ultra-sound, *PET/CT* Positron emission computed tomography, *CTA* Computed tomography angiography, *MRA* Magnetic resonance angiography

#### GCA-related cerebrovascular ischemic events

Among these 271 patients, 14 (5.2%) presented with GCA-related CIE at diagnosis or relapse (10 constituted and 4 transient), with a median delay between the GCA-related CIE and diagnosis or relapse of GCA of 5 days (− 22–0). Regarding the territory of these 14 CIE, 8 (57.2%) occurred in the vertebrobasilar territory, 5 (35.7%) in the carotid territory, and the remaining one associated multifocal ischemic and hemorrhagic strokes related to intra-cranial vasculitis. A detailed description of these GCA-related CIE is given in Additional file 1. Two of these patients presented a recurrent GCA-related CIE.

#### Comparison of GCA patients with and without GCA-related cerebrovascular ischemic events

Patients with GCA-related CIE had a lower body mass index (BMI) (22.5 vs 24.9 kg/m^2^, *p* = 0.02). Imaging studies showed that patients with GCA-related CIE more frequently had vertebral artery thrombosis on Doppler US (17% vs 0.8%, *p* = 0.012), vertebral artery involvement (50% vs 3.4%, *p* < 0.001), and intracranial artery involvement (50% vs 1.8%, *p* < 0.001) on CTA and/or MRA. Among the four patients who presented with vertebral artery thrombosis on Doppler US, vertebral thrombosis was confirmed by MRA in three cases. There was also a trend towards more frequent involvement of carotid artery on CTA and/or MRA with borderline significance (36% vs 15%, *p* = 0.053). There was more frequent axillary artery involvement on PET/CT (55% vs 20%, *p* = 0.016).

There was a trend towards less cranial symptoms in patients with GCA-related CIE (headache: 54% vs 72%, *p* = 0.2; scalp tenderness 15% vs 39%, *p* = 0.087). Patients with GCA-related CIE tended to be mostly men (57% vs 32%, *p* = 0.075) and smokers (54% vs 30%, *p* = 0.12), but the difference was not statistically significant. There was no statistical difference regarding the other traditional vascular risk factors (diabetes, arterial hypertension, dyslipidemia).

#### Treatment and ischemic vascular events during follow-up

Regarding the treatments received, patients with GCA-related CIE had more frequently been treated by glucocorticoids pulses (69% vs 25%, *p* = 0.001). They also tended to receive tocilizumab more often than the control group, but this result did not reach significance (29% vs 12%, *p* = 0.081). Among the twelve patients who presented with CIE at diagnosis of GCA, treatment was initiated at a median of 4 days (0–15.8).

During follow-up, 32 (12%) patients had an ischemic vascular event. Among the 14 patients with GCA-related CIE, 2 (14%) presented with a non GCA-related CIE during follow-up: 1 of cardioembolic cause, 1 related to small artery disease. Among the 257 patients of the control group, 30 (12%) presented with an ischemic vascular event, of which 17 (57%) were non GCA-related CIE, 9 (30%) myocardial infarction, and 4 (13%) limb ischemia. The etiology of the 17 non GCA-related CIE was available for 16 of them: 2 (11%) were atheromatous, 5 (33%) cardioembolic, 2 (17%) related to small artery disease, 1 (5.6%) of septic cause (embolism of endocarditis), and 6 (33%) cryptogenic strokes.

Among the population of patients with GCA-related CIE, 2 of them presented with non GCA-related CIE during follow-up, but this complication occurred later than in the control group (3 073 vs 1 378 days, *p* = 0.052).

Mortality tended to be higher in patients with GCA-related CIE, but with borderline significance (29% vs 11%, *p* = 0.075).

### Systematic review of the literature and meta-analysis

#### Study selection

**Fig. 1:**
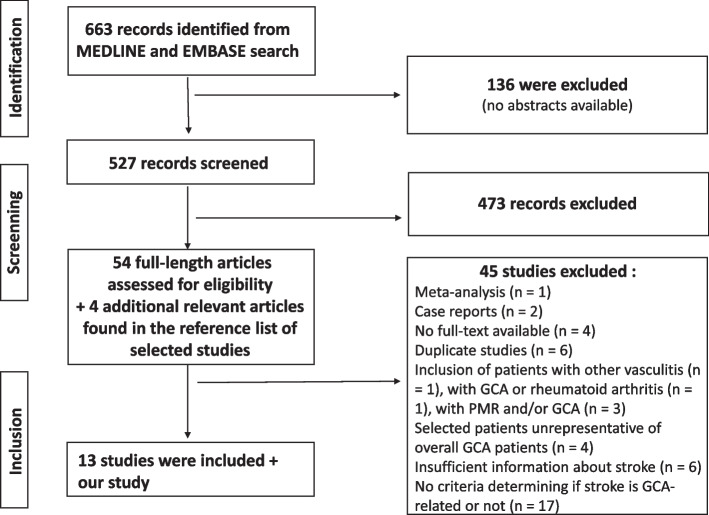
Flow chart showing search strategy to identify studies in the meta-analysis

A total of 663 potentially relevant articles were found from MEDLINE and EMBASE search, after exclusion of duplicated articles. After screening, 54 potentially eligible studies were selected, 4 relevant articles found in the reference list of selected studies were added, and full-text copies of these citations were obtained. Of these articles, 14 studies, including our cohort, were included in the meta-analysis, representing a total population of 3553 patients (Fig. 1). The main characteristics of the included studies are summarized in Table 2.

**Table 2:** Main characteristics of studies in the prevalence meta-analysis

First author	Country	Year	Study design	Study period	GCA diagnosis criteria	GCA-related cerebrovascular ischemic event (CIE) definition	Population	Number of GCA-related CIE	Prevalence
Berger [9]	Switzerland	2009	Retrospective	10 years (1997–2007)	Biopsy-proven GCA and/or ACR criteria (≥ 3/5)	If occurring within 2 weeks of diagnosis. TIA were excluded	85	2	2.35%
Cid [7]	Spain	1998	Retrospective	16 years	Biopsy-proven GCA	If they were concomitant with disease manifestations and in the absence of significant vascular risk factors such as heavy smoking, hypertension, hypercholesterolemia, or diabetes	200	3	1.50%
Coronel [18]	Spain	2021	Retrospective	11 years (2005–2016)	ACR criteria (≥ 3/5). Diagnosis based on the patient’s clinical history, blood tests and imaging results	If occurring during the first 4 weeks following diagnosis or relapse of GCA. CTA were run on all patients and the information of all MRA studies available was recorded, to rule out other etiologies such as atherosclerosis, arrhythmia, bleeding and others causes	123	9	7.32%
De Boysson [15]	France	2017	Retrospective	20 years (1995–2015)	ACR criteria (≥ 3/5)	If occurring at the time of diagnosis or within 4 weeks after starting GCA therapy. TIA were excluded	876	35	4.00%
Gonzalez-Gay [11]	Spain	2009	Retrospective	27 years (1981–2008)	Biopsy-proven GCA	If occurring between the onset of symptoms of the disease and 4 weeks after the onset of steroid therapy. All patients in whom stroke was diagnosed had lesions on CTA and/or MRA that were read by a neuroradiologist and correlated clinically by a neurologist. TIA were excluded	287	8	2.79%
Hočevar [20]	Slovenia	2020	Prospective	8 years (2011–2019)	Corresponding clinical and laboratory features, and a positive result of a TAB, or CDS or PET/CT	If occurring after the onset of GCA symptoms and up to 1 month after the initiation of glucocorticoid therapy. TIA were excluded	295	9	3.05%
Lee [13]	USA	2006	Retrospective	15 years (1989–2004)	ACR criteria (≥ 3/5)	If other signs, symptoms, or laboratory evidence of a recurrence was present. Hemispheric strokes only were included. TIA were excluded	143	6	4.20%
Narváez [16]	Spain	2008	Retrospective	18 years (1986–2004)	Biopsy-proven GCA and/or ACR criteria (≥ 3/5)	If occurring within the time between the onset of GCA symptoms and 4 weeks after the onset of corticosteroid therapy	121	5	4.13%
Nesher [19]	Israel	2004	Retrospective	20 years (1980–2000)	Biopsy-proven GCA and/or ACR criteria (≥ 3/5)	If occurring at presentation or within 2 weeks of GCA diagnosis. Strokes developing later were considered GCA related only when associated with at least 1 of the other GCA-related signs or symptoms, or laboratory evidence of acute-phase reaction. TIA were excluded	175	19	10.86%
Pariente [17]	France	2019	Retrospective	8 years (2010–2018)	ACR criteria (≥ 3/5)	If occurring within a delay between GCA diagnosis and stroke inferior to 12 months, and with no other etiology of stroke, notably the absence of atrial fibrillation at the time of stroke	139	18	12.95%
Parreau [14]	France	2022	Retrospective	38 years (1982–2020)	ACR criteria (≥ 3/5). In biopsy-negative cases, if at least 3 criteria fulfilled, or if only 2 criteria fulfilled with PET/CT or CTA strongly suggestive of vasculitis	If occurring at the time of diagnosis or within 4 weeks of starting GCA treatment. If the neurological event preceded GCA diagnosis, a review of neurovascular imaging charts allowed to recognize retrospectively GCA-related CIE. Strokes without concurrent atrial fibrillation occurring during GCA flare were also regarded as GCA-related. TIA were excluded	560	19	3.39%
Penet (present study)	France	2022	Retrospective	11 years (2010–2020)	ACR criteria (≥ 3/5)	If it was clearly linked to GCA at diagnosis or relapse after reviewing patient's medical records, in the absence of another well identified etiology (mainly atherosclerosis, embolic or cerebral small vessel disease)	271	14	5.17%
Salvarani [8]	Italy	2009	Retrospective	19 years (1986–2005)	Biopsy-proven GCA	If occurring within the time between the onset of GCA symptoms and 4 weeks after the onset of corticosteroid therapy	180	5	2.78%
Zenone [10]	France	2013	Retrospective	12 years (1999–2012)	ACR criteria (≥ 3/5)	Doppler US or MRA of the supra-aortic vessels performed in order to demonstrate concentric segmental narrowing, stenosis and/or occlusions suggestive of vasculitis	98	6	6.12%

*GCA* Giant cell arteritis, *CIE* Cerebrovascular ischemic events, *TIA* Transient ischemic attack, *TAB* Temporal artery biopsy, *ESR* Erythrocyte sedimentation rate, *CRP* C-reactive protein, *CDS* Color Doppler ultra-sound, *CTA* Computed tomography angiography, *MRA* Magnetic resonance angiography, *PET/CT* Positron emission computed tomography

#### Cerebrovascular ischemic events prevalence meta-analysis

**Fig. 2:**
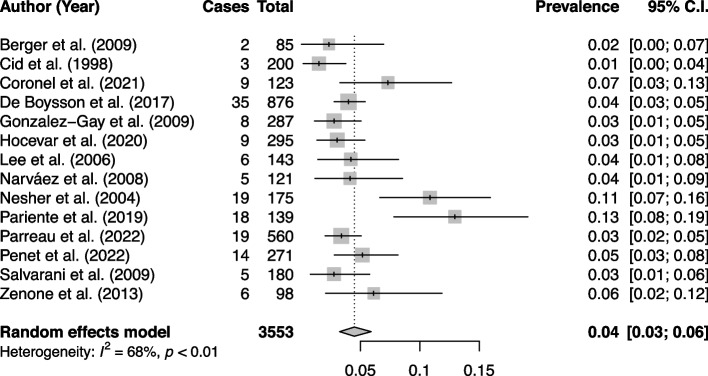
Forest plot of GCA-related CIE prevalence in all studies

The pooled prevalence of GCA-related CIE for all studies was 0.04 [(95% CI 0.03–0.06); *I*^*2*^ = 68%; *p* (het) < 0.01] (Fig. 2).

Outlier and influential analyses were used to explain heterogeneity. After identification of Pariente et al. as outlier study, the resulting pooled prevalence was 0.04 (95% CI 0.03–0.05) with a decrease in heterogeneity (*I*^*2*^ = 55%).

Moreover, we attempted to explain heterogeneity by distinguishing studies having used a delay cut-off of 4 weeks from diagnosis to define a CIE to be GCA-related. Meta-regression using this delay cut-off as moderator did not explain heterogeneity (*p* = 0.25). Hence, no subgroup analysis was conducted.

## Discussion

Relating a CIE to GCA can be challenging because of multiple other CIE causes that patients may present. The aim of our study was to evaluate the prevalence of GCA-related CIE in our cohort using strict criteria along with a systematic review and meta-analysis of the literature, to determine the pooled prevalence of CIE in GCA. We further characterized patients with GCA-related CIE in our well-phenotyped cohort with various imaging modalities available. The main results of our study are as follows: (i) a prevalence of GCA-related CIE of 5.2% in our cohort, (ii) a pooled prevalence of GCA-related CIE of 4% (95% CI 3–6%) in our meta-analysis of the literature, including our new cohort, (iii) an association between GCA-related CIE and lower BMI, vertebral and intracranial arteries involvement on Doppler US or CTA/MRA, and axillary arteries involvement on PET/CT.

### GCA-related CIE prevalence

There is considerable heterogeneity about the definition of a GCA-related CIE between the different studies, leading to an uncertainty regarding the actual prevalence of CIE, which can range from 1.5% [7] to 12.95% [17]. Our meta-analysis found a pooled prevalence of GCA-related CIE of 4% (95% CI 3–6%, *I*^*2*^ = 68%). The heterogeneity decreased to 55% after identifying Pariente et al. as an outlier study. The high prevalence of 12.95% in Pariente et al. could be partly explained by a long delay of up to 12 months from diagnosis in defining GCA-related CIE [17]. In 11 out of 14 studies included in our meta-analysis, a temporal criterion of up to 4 weeks delay from diagnosis/relapse of GCA to CIE was used. However meta-regression using this delay cut-off as moderator did not explain heterogeneity (*p* = 0.25). In our experience, to relate a CIE to GCA, a complete lookup of all clinical, laboratory, and imaging data is needed to evaluate the activity of the vasculitis, in addition to eliminating other usual causes of CIE.

### GCA-related CIE risk factors

#### Vascular risk factors, clinical and biological features

The role of traditional vascular risk factors in GCA-related CIE remains controversial. Some authors found an association with CIE [6, 8, 11, 17, 22, 26], whereas others did not show any significant association [10, 13, 15, 19, 21]. High prevalence of vascular risk factors in aged patients with GCA may be a confounding factor. In our study, GCA-related CIE were not significantly associated with vascular risk factors except for BMI which was lower in GCA-related CIE patients. High BMI has been reported by Tomasson et al. to protect against the occurrence of GCA, especially in women [29]. A meta-analysis by Ungprasert et al. also found a statistically significant inverse relationship between BMI and risk of subsequent development of GCA [30]. However, we did not find in the literature any association between BMI and CIE in GCA.

Regarding clinical features, only jaw claudication has been linked to CIE by González-Gay et al. [4] with an OR of 3.49 (95% CI 0.63–19.2, *p* = 0.151), but this association was not found in other studies. In a study by Hočevar et al., jaw claudication was a risk factor for visual and cerebrovascular ischemic complications when studied as a combined endpoint (OR 3.43, 95% CI 1.84–6.42, *p* < 0.001), but this remained a risk factor only for visual complications in multivariable logistic regression performed to determine predictors separately for visual complications and stroke [20]. Moreover, Gonzalez-Gay et al. found in a 2009 study a reduced risk of stroke in patients who complained of headache at the time of GCA diagnosis (OR 0.15, 95% CI 0.02–0.99, *p* = 0.05) [11]. Parreau et al. also found significantly less often headache and scalp tenderness among patients with GCA-related CIE [14]. This is consistent with our results, showing a trend towards less cranial symptoms (headache and scalp tenderness) in patients with GCA-related CIE.

A high clinical and biological inflammatory activity as a protective factor against visual and cerebrovascular ischemic complications was first reported by Cid et al. [7] and then has been consistently found among different studies. Patients presenting fever, constitutional symptoms, polymyalgia rheumatica, high CRP and ESR levels, and low hemoglobin had a reduced risk of both visual and cerebrovascular complications, whether these complications are studied as a combined endpoint [4, 7–9, 13, 19, 20, 23, 24], or whether only CIE are studied [11, 15, 26]. A possible explanation of this association was given by Hernández-Rodríguez et al., who demonstrated a lower tissue expression and circulating level of the inflammatory cytokine IL-6 in GCA patients with ischemic complications, which have a pro-angiogenic effect that could be a compensatory mechanism for ischemia in GCA [31]. However, our study, as well as Pariente et al. [17], did not show any significant association between these inflammatory clinical or biological characteristics and CIE.

#### Ophthalmologic ischemic complications

Ophthalmic ischemic complications have also been reported as a predictor of CIE in some studies. Gonzalez-Gay et al. [4, 11] found that the best predictor for the occurrence of stroke was the presence of permanent visual loss (OR 7.65, 95% CI 1.58–37.0, *p* = 0.012) as well as De Boysson et al. (OR 5, 95% CI 2.14–12.33, *p* = 0.0002) [15]. However, our study and others [10, 21, 26] did not find significant association between ophthalmologic and cerebrovascular ischemic complications.

#### Morphological imaging (Doppler US, CTA, MRA)

GCA-related CIE more often involve the vertebrobasilar territory (40–60%) rather than the carotid territory, whereas among atheromatous cerebrovascular events, only 15–20% occur in vertebrobasilar territory [2, 32]. Our results are consistent with the literature, 8 (57.2%) of our 14 GCA-related CIE occurring in the vertebrobasilar territory.

In our study, we found an association between GCA-related CIE and vertebral artery thrombosis on Doppler US (17% vs 0.8%, *p* = 0.012) and vertebral artery involvement on CTA and/or MRA (50% vs 3.4%, *p* < 0.001). Vertebral arteries involvement is known to be frequent in GCA, and the usefulness of Doppler ultrasound for the detecting vertebral involvement is already well established [33, 34]. In a systematic review of the literature, Elhfnawy et al. found that multiple stenoses/occlusions in the vertebrobasilar territory affected around 70% of stroke patients with GCA [35].

Involvement of intracranial vessels has been historically considered exceptional because they contain few or no internal elastic laminae and do not contain vasa vasorum [32, 36]. But recent literature has shown that intracranial vessel involvement is more frequent than previously thought, with the development of more advanced imaging techniques including 3 T MRI. The most affected intracranial arteries are first internal carotid, followed by intradural vertebral arteries part (V4) and posterior cerebral arteries, and these patients have high incidence of CIE and poor prognosis [37–39]. In a prospective study, Siemonsens et al. used 3 T MRI with fat-saturated T1WI pre- and post-contrast application optimized for assessment of intradural vessel wall enhancement and found that, among 20 GCA patients, 10 (50%) presented intradural internal carotid artery involvement, 9 presented vertebral arteries involvement, 5 of them bilateral. One patient presented middle cerebral artery involvement. However, intradural vessels involvement did not correlate with intracranial steno-occlusive lesions nor cerebral infarction; thus, its prognostic value remained uncertain [40]. Our study confirms the high prevalence of intracranial arteries involvement among GCA patients who presented with CIE, with 7 (50%) of our 14 patients with GCA-related CIE presenting intracranial arteries involvement on brain MRA and/or CTA, but the clinical-radiological correlation was not constant. Moreover, we identified a significant association between intracranial arteries involvement on CTA and/or MRA and GCA-related CIE (50% vs 1.8%, *p* < 0.001).

These findings and our results support the fact that GCA-related CIE can also occur in non-vertebrobasilar territory and that high performance MRI can be useful in this context. Further research is needed to better define the prognostic value of vertebral and intracranial arteries involvement and its predictive potential for the occurrence of CIE.

#### Functional imaging (PET/CT)

Axillary arteries are among the most often involved arteries in GCA. Kermani et al. found that the most frequently affected arteries on morphological imaging in GCA were subclavian (42%) and axillary (32%) in their prospective cohort of 187 patients [41]. Blockmans et al. found that the most affected arteries on PET/CT at diagnosis were subclavian (74%), abdominal (54%) and thoracic (51%) aorta, and axillary (40%) in their prospective cohort of 35 patients [42]. In our study, we found an association between axillary arteries involvement on PET/CT and GCA-related CIE (55% vs 20%, *p* = 0.016). This result should be interpreted with caution considering that axillary arteries are the easiest to distinguish on PET/CT compared to other supra-aortic trunks, whose interpretation may be disturbed by artifacts.

Few studies have evaluated the risk of ischemic complications according to PET/CT findings. In a prospective study by Mestre-Torres et al., 30 GCA patients, of which 21 presenting ischemic complications at diagnosis (mainly ophthalmologic, only one patient had CIE), underwent PET/CT during the first 10 days of steroid therapy. Patients with ischemic manifestations showed vertebral artery hypermetabolism on PET/CT more frequently than patients without, although the difference did not reach statistical significance (OR 5, 95% CI 0.99–24.86, *p* = 0.051). The involvement of all other territories on PET/CT was found protective against ischemic complications, including aorta (OR 0.05, 95% CI 0.008–0.36, *p* = 0.001) and axillary arteries (OR 0.08, 95% CI 0.01–0.49, *p* = 0.004) [43].

Comparison of these results with our findings is difficult as our primary endpoint consisted exclusively of CIE. Moreover, in our larger cohort, 171 patients underwent PET/CT with a median delay from steroid initiation of 1 day (− 7–20). Further studies are needed to assess the usefulness of the PET/CT for evaluating the risk of ischemic events, especially CIE.

### Ischemic vascular events during follow-up and mortality

Among our patients with GCA-related CIE, 2 of them presented a non GCA-related CIE during follow-up, but this complication occurred later than in the control group. This might be explained by tighter follow-up and a better control of cardiovascular risk factors in these high-risk patients on corticosteroid therapy.

Patients with GCA-related CIE presented a shorter follow-up period in our cohort. This may be explained by a higher mortality in this group. Indeed, we observed a non-significant trend toward a higher mortality in patients with GCA-related CIE (29% vs 11%, *p* = 0.075), which is consistent with the results of Pariente et al. who showed that the overall survival was significantly decreased in GCA patients with stroke [17].

### Treatment

In our study, patients with GCA-related CIE tended to receive tocilizumab more often than the control group, with borderline significance (29% vs 12%, *p* = 0.081). In 4 patients, GCA-related CIE motivated the prescription of tocilizumab (2 at a relapse, 2 immediately associated with corticosteroids at diagnosis). Interestingly, none of the patients who were prescribed tocilizumab experienced recurrent CIE after the introduction of the immunosuppressant, although one of them with severe multifocal strokes died within 3 months after diagnosis from stroke complications. However, the design of our study does not allow us to draw conclusions about the potential protective effect of tocilizumab against CIE, and further research is needed to determine the usefulness of tocilizumab in GCA-related CIE.

### Strengths and limitations

The major strengths of our study are a large cohort of 271 well-phenotyped GCA patients, the use of strict criteria to define a CIE to be GCA-related, and the availability of detailed clinical, laboratory, and various imaging modalities characteristics. Our systematic review and meta-analysis of the literature allowed us to estimate the pooled prevalence of GCA-related CIE. Although our study has shed the light on new associations between patients’ imaging characteristics and GCA-related CIE, no multivariate analysis could be performed mainly because each patient did not undergo the complete set of these different imaging modalities. Further studies are needed to confirm risk factors of CIE in GCA. Unfortunately, because of the retrospective nature of our study, functional outcome scores such as Barthel index and Rankin scale were not calculated. These scores would have allowed us to compare outcomes between groups, improve the quality of care of our patients and further shed insight on the benefits of corticosteroids in addition to standard of care for GCA-related CIE.

## Conclusion

CIE are among the most severe ischemic complications of GCA, with an uncertainty regarding their real prevalence, due to the difficulties relating them to GCA. According to our meta-analysis of the literature, the pooled prevalence of GCA-related CIE is 4%. Because GCA-related CIE increase morbidity and mortality, identifying their risk factors is crucial to prevent their occurrence and improve patients’ prognosis. Our study highlights new associations between various imaging modalities features and GCA-related CIE, such as vertebral artery thrombosis on Doppler US, vertebral artery involvement and intracranial artery involvement on CTA and/or MRA, and axillary artery involvement on PET/CT. Although lower BMI had been previously reported to be associated with occurrence of GCA, we report for the first time its association with GCA-related CIE. Further research is needed to confirm these associations as risk factors. Finally, although the use tocilizumab was motivated by the occurrence of GCA-related CIE in 4 of our patients with successful results, further studies are needed to establish its use in the prevention of GCA-related CIE.

## Supplementary Information

**Additional file 1. Table S1.** Cerebrovascular ischemic events (CIE) characteristics.

## Data Availability

The datasets used and/or analyzed during the current study are available from the corresponding author on reasonable request.

## Abbreviations

GCA: Giant cell arteritis
CIE: Cerebrovascular ischemic events
ICD-10: International Classification of Diseases, 10th Edition
ACR: American College of Rheumatology
TAB: Temporal artery biopsy
Doppler US: Doppler ultrasound
PET/CT: Positron emission computed tomography
CTA: Computed tomography angiography
MRI: Magnetic resonance imaging
MRA: Magnetic resonance angiography
MOOSE: Meta-analysis of Observational Studies in Epidemiology
JBI: Joanna Briggs Institute
SD: Standard deviation
IQR: Interquartile range
CRP: C-reactive protein
BMI: Body mass index
TIA: Transient ischemic attack
ESR: Erythrocyte sedimentation rate
95% CI: 95% Confidence interval
