# Supplementary material for: Giant cell arteritis-related cerebrovascular ischemic events: a French retrospective study of 271 patients, systematic review of the literature and meta-analysis
Source: Arthritis Res Ther. 2023 Jul 7;25:116. doi: 10.1186/s13075-023-03091-x (PMC10326952; doi:10.1186/s13075-023-03091-x)
Supplement: Supplementary file 1 — Additional file 1. Table S1. Cerebrovascular ischemic events (CIE) characteristics. [file 13075_2023_3091_MOESM1_ESM.docx]

Table S1. Cerebrovascular ischemic events (CIE) characteristics

| Case | Age (years) | First CIE delay to diagnosis/relapse (days) | First CIE occurrence (diagnosis or relapse) | First CIE territory | Treatment initiation delay from first CIE (days) | Treatment | Recurrent CIE territory | Treatment changes |
| --- | --- | --- | --- | --- | --- | --- | --- | --- |
| 1 | 77 | -15 | Diagnosis | Vertebrobasilar: multiple bilateral cerebellar and pontine ischemic lesions | 4 | Corticosteroids 1 mg/kg/d without pulse | Vertebrobasilar: right paramedian pontine ischemic stroke, with bilateral PET hyperfixation of vertebral arteries, and circumferential contrasting of basilar stem and right vertebral artery on MRA, indicative of a GCA relapse 8 month after diagnosis | Initiation of TCZ and reascending corticosteroids at 1 mg/kg/d |
| 2 | 81 | 0 | Relapse at 19 months of diagnosis while steroid tapering | Carotidian: right lenticulo-caudal stroke | -952 | Reascending corticosteroids at 1 mg/kg/d | 0 |  |
| 3 | 63 | -2 | Diagnosis | Carotidian TIA: transient monocular visual loss of the right eye | 0 | Corticosteroids 1 mg/kg/d preceded by steroid pulse | 0 |  |
| 4 | 86 | -51 | Diagnosis | Vertebrobasilar: bilateral multiple ischemic lesions in the vertebrobasilar territory | 54 | Corticosteroids 1 mg/kg/d without pulse | 0 |  |
| 5 | 68 | 0 | Relapse at 14 months of diagnosis while steroid tapering | Vertebrobasilar TIA: mouth deviation and dysarthria | -488 | Initiation of MTX and reascending corticosteroids at 1 mg/kg/d | 0 |  |
| 6 | 63 | -6 | Diagnosis | Intracranial cerebral vasculitis: 2 hemorrhagic strokes in the right frontal and occipital lobes, disseminated punctiform cortical ischemic lesions, and a small meningeal hemorrhage in the right frontal region | 6 | TCZ immediately associated with corticosteroids 1 mg/kg/d preceded by steroid pulse | 0 |  |
| 7 | 93 | -4 | Diagnosis | Vertebrobasilar: multiple bilateral ischemic lesions in the vertebrobasilar territory | 4 | Corticosteroids 1 mg/kg/d preceded by steroid pulse | 0 |  |
| 8 | 89 | -24 | Relapse 11 years after discontinuation of corticosteroids | Vertebrobasilar: Right anterolateral bulbar stroke and right middle cerebellar stroke | 3 | Resumption of corticosteroids 1 mg/kg/d preceded by steroid pulse | 0 |  |
| 9 | 63 | 0 | Diagnosis | Vertebrobasilar: multifocal strokes in the territory of the posteroinferior cerebellar arteries and bilateral posterior cerebral arteries | 0 | Corticosteroids 1 mg/kg/d preceded by steroid pulse | 0 |  |
| 10 | 66 | 5 | Diagnosis | Multiple carotidian TIAs: bilateral tilting transient monocular blindness on day 2 of corticosteroid therapy, with bilateral inflammatory carotid stenosis | -1 | Corticosteroids 1 mg/kg/d preceded by steroid pulse | 0 |  |
| 11 | 77 | -24 | Diagnosis | Vertebrobasilar: ischemic lesions in the territory of the left posterior cerebral artery: left parieto-occipital and thalamic lesions | 24 | Corticosteroids 1 mg/kg/d without pulse | 0 |  |
| 12 | 74 | 1 | Diagnosis | Carotidian: multifocal strokes in the right sylvian territory on day 2 of corticosteroid therapy, with bilateral severe involvement of both carotid, vertebral and intra-cranial cerebral arteries on brain MRA and CTA | -1 | Corticosteroids 1 mg/kg/d preceded by steroid pulse | Carotidian: recurrence of stroke within the first month after diagnosis in the right anterior cerebral artery territory, then multifocal strokes in left carotidian territory | Initiation of TCZ. Rapid death of the patient during follow-up |
| 13 | 60 | -27 | Diagnosis | Carotidian: Superficial right sylvian stroke with occlusion of the right suprabulbar internal carotid artery and stenosis of the left intracavernous internal carotid artery | 26 | TCZ immediately associated with corticosteroids 1 mg/kg/d preceded by steroid pulse | 0 |  |
| 14 | 82 | -13 | Diagnosis | 3 vertebrobasilar TIAs: linked to GCA because of P2 stenosis of the left posterior cerebral artery on MRA | 13 | Corticosteroids 1 mg/kg/d without pulse | 0 |  |

*CIE* Cerebrovascular ischemic event, *TIA* Transient ischemic attack, *MTX* methotrexate, *TCZ* tocilizumab, *MRA* Magnetic resonance angiography, *CTA* Computed tomography angiography, *PET* Positron emission tomography.
